# Hierarchical likelihood opens a new way of estimating genetic values using genome-wide dense marker maps

**DOI:** 10.1186/1753-6561-5-S3-S14

**Published:** 2011-05-27

**Authors:** Xia Shen, Lars Rönnegård, Örjan Carlborg

**Affiliations:** 1 The Linnaeus Centre for Bioinformatics, Uppsala University, Uppsala, Sweden; 2 Statistics Unit, Dalarna University, Borlänge, Sweden; 3 Department of Animal Breeding & Genetics, Swedish University of Agricultural Sciences, Uppsala, Sweden

## Abstract

**Background:**

Genome-wide dense markers have been used to detect genes and estimate relative genetic values. Among many methods, Bayesian techniques have been widely used and shown to be powerful in genome-wide breeding value estimation and association studies. However, computation is known to be intensive under the Bayesian framework, and specifying a prior distribution for each parameter is always required for Bayesian computation. We propose the use of hierarchical likelihood to solve such problems.

**Results:**

Using double hierarchical generalized linear models, we analyzed the simulated dataset provided by the QTLMAS 2010 workshop. Marker-specific variances estimated by double hierarchical generalized linear models identified the QTL with large effects for both the quantitative and binary traits. The QTL positions were detected with very high accuracy. For young individuals without phenotypic records, the true and estimated breeding values had Pearson correlation of 0.60 for the quantitative trait and 0.72 for the binary trait, where the quantitative trait had a more complicated genetic architecture involving imprinting and epistatic QTL.

**Conclusions:**

Hierarchical likelihood enables estimation of marker-specific variances under the likelihoodist framework. Double hierarchical generalized linear models are powerful in localizing major QTL and computationally fast.

## Background

Genetic analyses in livestock studies are generally based on information from pedigrees and molecular markers. Traditionally, a kinship matrix can be calculated using the pedigree data, which can be used in a *generalized linear mixed model* (GLMM) to estimate breeding values. By including genetic marker information, *genomic estimated breeding values* (GEBV) can be obtained taking into account the information from these markers, and also *quantitative trait loci* (QTL) can be mapped by associating genotypes at a certain locus to the phenotype observations.

Dense marker genotypes along genome can now be affordably obtained due to new and efficient methods for typing *single nucleotide polymorphism* (SNP) markers. The dense SNP maps have made *genome-wide association* (GWA) studies popular for gene detection. Classic GWA methods [1], commonly applied to study genetic diseases in humans, are based on simple repeated single marker tests across the genome. To achieve more powerful mapping and better prediction, a unified model including all the SNPs in the genome is preferred. Such models have been estimated using Bayesian methods, implemented by Markov chain Monte Carlo (MCMC) techniques that are computationally demanding [2-5]. Lee and Nelder developed the double hierarchical generalized linear model (DHGLM) in the likelihoodist framework [6]. DHGLM enables estimation of marker-specific variances using a fast iterative algorithm without specifying any prior distributions [7]. The likelihoodist way of estimation is conducted through a likelihood function named *hierarchical likelihood* (*h*-likelihood) [8].

The aim of this paper is to map QTL and report GEBV for the simulated dataset provided by QTLMAS 2010 workshop. We employ a unified analysis via the *h*-likelihood and model the data using DHGLM. GEBV are calculated from the estimated marker effects, and QTL are mapped by the estimated marker-specific variances.

## Methods

### Data

The dataset used in this paper was simulated for the QTLMAS 2010 workshop (Poznań, Poland). A pedigree consisting of 3226 individuals in 5 generations (*F*_0_ - *F*_4_) was simulated, where *F*_0_ contains 5 males and 15 females. Each female was mated once and gave birth to about 30 progeny. Two traits were simulated, where one is quantitative (QT), and the other is binary (BT). Young individuals in *F*_4_ (individuals 2327 to 3226) had no phenotypic records. The genome was assumed to be about 5 × 10^8^ bp long, consisting of 5 chromosomes, each of which contained about 1 × 10^8^ bp. Each individual was genotyped for 10031 biallelic SNPs in the genome.

### Models

DHGLM provides a unified analysis for both QTL mapping and genomic breeding value estimation. Similar to BayesA, the data are modeled on two levels, i.e. both the phenotypic mean and the variance are modeled with random effects. For a quantitative trait, the phenotype **y** (*n* × 1 vector) is postulated as a random effect model

**y** = **X*β*** + **Zg** + **e** (1)

where **g** ~ ***N***(**0**, diag(**λ**)) are the SNP effects, **λ** = (λ_1_, λ_2_,..., λ_m_)′ are the variances of the SNP effects, and the residuals **e** ~ *N*(0, *σ*^2^**I**). The fixed effects ***β*** included an intercept and the sex effect in our application to reduce the residual errors. The SNP variances **λ** are modeled as

log **λ** = **1***a* + **b** (2)

with an intercept *a* and normally distributed random effects **b**. The *genomic estimated breeding value* (GEBV) for individual *i* is computed as . QTL can be scanned using the marker-specific variances **λ**. For a binary trait, the mean of **y**, is modeled by the same linear predictor **X*β*** + **Zg** through a logit link function.

For the marker-specific variances, the correlated random effects, **b**, follow a multivariate normal distribution with a mean of zero and a variance-covariance matrix , where *m* is the number of SNPs and *k*, *l* are the SNP indices. When *ρ* = 1, all the SNPs have a constant variance (GLMM); when *ρ* = 0, the SNPs are assumed to be independent (DHGLM); and for 0 <*ρ* < 1, the correlation between two SNPs is a monomial function of *ρ*, which is referred to as the *smoothed* DHGLM [10]. We propose the use of smoothed DHGLMs since it reduces the noise in marker-specific variance estimates and highlights the signals of QTL. *ρ*, regarded as a spatial correlation parameter, was chosen to be 0.9 in this paper, which nicely shrank the SNPs with zero effect.

The overall phenotypic variance can be expressed as(3)

where  is the variance of **z***._j_* (the *j*-th column of **Z**) across individuals. These variance values can be directly calculated from the data. The contribution (heritability) of a particular SNP is expressed by [4].

### Fitting algorithm

According to the extended likelihood principle, inference of the random SNP effects **g** should be drawn through the *h*-likelihood, fixed effects ***β*** through the marginal likelihood, and variance components **λ**, *σ*^2^ and  through the adjusted profile likelihood [11]. However, for efficient estimation, we propose to initialize variance components and iterate the following steps until convergence [7],

• Solve the following WLS problem for  and **ĝ**,(4)

Where  and . The subscript *M* stands for ‘mean’.

• Update *σ*^2^ by fitting the deviance residuals  using an intercept-only gamma GLM and prior weight **w***_M_* = (**1** – **q***_M_*)/2, where  are the residuals of (4), and  are the diagonal elements of  The subscript 1 and 2 stand for individuals (1 to *n*) and SNPs (*n* + 1 to *n* + *m*), respectively.

• Solve the following WLS problem for *â* and ,(5)

where , , **z** = log **λ** + (**d**_***M***2_ – **λ**)/**λ** is linearized **λ** in a gamma GLM with a log link, and **L** satisfies **LL**′ = **A**. The subscript *D* stands for ‘dispersion’.

• Update  by fitting the deviance residuals  using an intercept-only gamma GLM and prior weight **w***_D_* = (**1** – **q***_D_*)*/*2, where ê*_D_* are the last *m* residuals of (5), and **q***_D_* are the last *m* diagonal elements of .

## Results and Discussion

### Estimation of SNP effects

**Figure 1:**
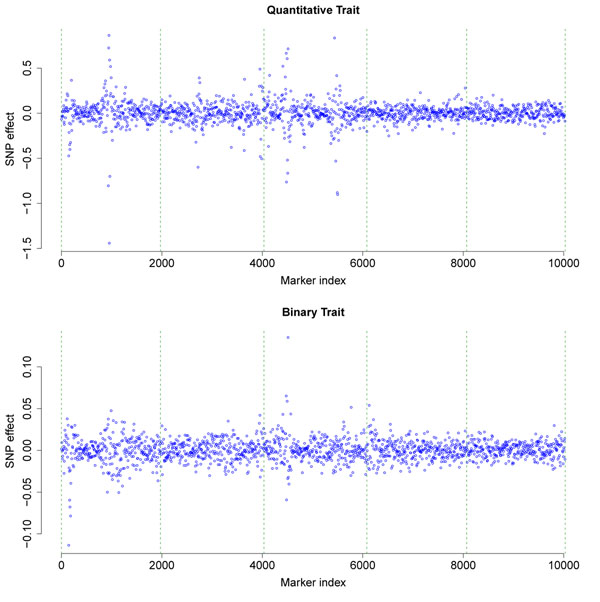
**Estimated SNP effects** The SNP effects were estimated using the smoothed DHGLM with spatial correlation parameter *ρ* = 0.9. The dashed vertical lines indicate the chromosome borders.

The effect of each SNP was estimated by a smoothed DHGLM with spatial correlation parameter *ρ* = 0.9 for both traits (Figure 1). For both traits, DHGLM shrank the estimated SNP effects for the loci not linked to main QTL towards zero; meanwhile, the SNPs linked to QTL were highlighted. Note that the extent of shrinkage depends on the spatial correlation parameter *ρ. ρ* = 0.9 was specified in our analyses since it produced better shrinkage and smoothing results for this particular dataset.

### QTL mapping

**Figure 2:**
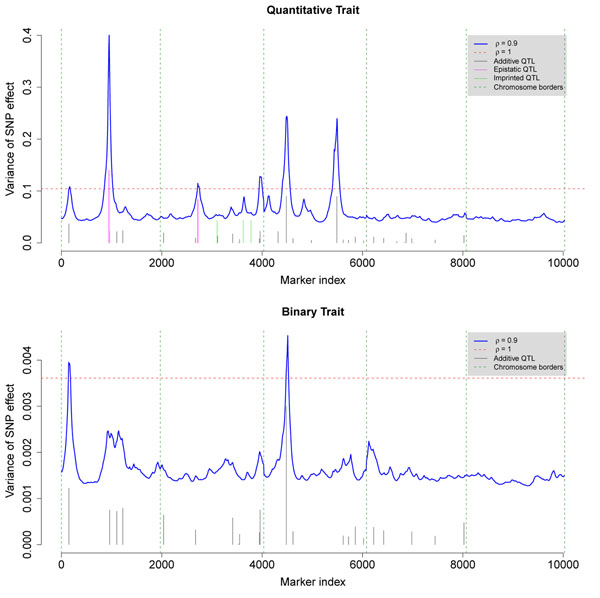
**QTL detection using estimated marker-specific variances** The marker-specific variances were estimated using the smoothed DHGLM with spatial correlation parameter *ρ* = 0.9. The dashed horizontal line is the overall variance of SNP effects estimated by GLMM. The peaks higher than this line were detected as QTL, and other small peaks below were suggestive QTL. Simulated QTL are also shown as vertical bars with their heights proportional to the variances they explained. For nice visualization, simulated variances are 1/50 magnified for QT and 1/1500 magnified for BT.

Moving from the mean part to the variance (dispersion) part of the models, marker-specific variances were estimated and used to detect QTL (Figure 2). The overall variance component estimate from GLMM can be regarded as a reference value (smoothed DHGLM with ρ = 1), which was estimated using the **hglm** package [12] in R [13]. The 6 peaks for QT, corresponding to SNP number 163, 952, 2719, 3957, 4493 and 5492, were QTL which had values greater than the overall variance component estimate. The two strong QTL for BT had similar positions as two for QT. Other small peaks lower than the reference line were suggestive QTL. Simulated main QTL were precisely mapped. The two main epistatic QTL pairs for QT were detected as two single QTL due to the very short distance between interacting SNPs. Heritability for QT and BT was calculated for detected QTL and suggestive QTL (Table 1). 30.35% and 33.42% of the phenotypic variance were explained for QT and BT, respectively. Phenotypes of QT and BT are significantly correlated with a Spearman’s rank correlation coefficient of 0.2431. However, joint-modeling both traits were not considered in this paper.

**Table 1:** Estimated heritability of the detected QTL and suggestive QTL for QT and BT.

	Chromosome	Position (bp)	*h*^2^ of QT	*h*^2^ of BT
QTL	1	8396357	0.0106	0.0957
	1	49965266	0.1096	-
	2	32741451	0.0167	-
	2	95418368	0.0177	-
	3	22590128	0.0606	0.1101
	3	71794627	0.0589	-
Suggestive QTL	1	49965266	-	0.0859
	2	79212967	0.0093	-
	2	95418368	-	0.0096
	3	4590043	0.0109	-
	3	39652617	0.0092	-
	3	84974466	-	0.0066
	4	1456752	-	0.0265

Sum			0.3035	0.3342

### GEBV

**Figure 3:**
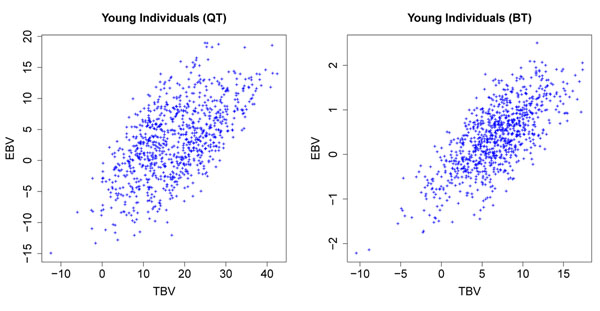
**Scatterplots of GEBV against TBV for the young individuals without phenotypic records** The GEBV were estimated using the smoothed DHGLM with spatial correlation parameter *ρ* = 0.9. The values are not scaled on the same mean.

GEBV were estimated for all the 3226 individuals in the pedigree. Examining out-sample prediction, we compare the GEBV with the true breeding values (TBV) for the young individuals (2327-3226) without phenotypic records (Figure 3). The correlation coefficients between GEBV and TBV were 0.60 for QT and 0.72 for BT. The linear regression slopes were 0.41 for QT and 0.62 for BT. Accuracy of GEBV was worse for QT than for BT mainly because three imprinted QTL were simulated only for QT, and QT had a more complicated genetic architecture.

## Conclusions

DHGLM were shown to be an efficient and reliable approach for both QTL mapping and genomic selection. Since DHGLM can be estimated by iterating interlinked GLMs, the execution time is greatly shortened comparing to the Bayesian computation. On a Macintosh laptop with a 2 GHz processor and 4 GB memory (1067 MHz), it took about 10-20 minutes, depending on starting values, to obtain our results using our implementation in R. No priors are required for parameters in DHGLM. Main QTL mapped via DHGLM showed very good accuracy though some QTL with small effects were shrunk or smoothed down. An R package **iQTL** has been implemented and is available on R-Forge: https://r-forge.r-project.org/R/?group id=845.

## List of abbreviations used

bp: base pair; DHGLM: double hierarchical generalized linear model; DNA: deoxyribonucleic acid; GEBV: genomic estimated breeding values; GLM: generalized linear model; GLMM: generalized linear mixed model; GWA: Genome-wide association; *h*-likelihood: hierarchical likelihood; HGLM: hierarchical generalized linear model; MCMC: Markov chain Monte Carlo; QTL: quantitative trait locus/loci; QTLMAS: quantitative trait loci and marker assisted selection; REML: restricted maximum likelihood; SNP: single nucleotide polymorphism; TBV: true breeding values; WLS: weighted least squares.

## Competing interests

No competing interest to declare by any of the authors.

## Authors contributions

XS, LR and ÖC initiated the study. XS analyzed the simulated common dataset of the QTLMAS 2010 workshop and drafted the paper. LR initiated the smoothed version of double hierarchical generalized linear models. XS, LR and ÖC worked on the revision together and approved the final manuscript.

## References

[B1] Cantor RM Lange K Sinsheimer JS Prioritizing GWAS results: A review of statistical methods and recommendations for their application Am 2010 86 6 22 10.1016/j.ajhg.2009.11.017 20074509 PMC2801749

[B2] Meuwissen THE Hayes BJ Goddard ME Prediction of total genetic value using genome-wide dense marker maps Genetics 2001 157 4 1819 1829 11290733 10.1093/genetics/157.4.1819 PMC1461589

[B3] Xu S Estimating polygenic effects using markers of the entire genome Genetics 2003 163 789 801 12618414 10.1093/genetics/163.2.789 PMC1462468

[B4] Xu S An empirical Bayes method for estimating epistatic effects of quantitative trait loci Biometrics 2007 63 513 521 10.1111/j.1541-0420.2006.00711.x 17688503

[B5] Yi N Xu S Bayesian LASSO for quantitative trait loci mapping Genetics 2008 179 1045 1055 10.1534/genetics.107.085589 18505874 PMC2429858

[B6] Lee Y Nelder JA Double hierarchical generalized linear models (with discussion) Applied Statistics 2006 55 139 185

[B7] Lee Y Nelder JA Pawitan Y Generalized linear models with random effects: unified analysis via h-likelihood 2006 Chapman & Hall/CRC

[B8] Lee Y Nelder JA Hierarchical generalized linear models (with discussion) J. R. Statist. Soc. B 1996 58 619 678

[B9] Yi N Banerjee S Hierarchical generalized linear models for multiple quantitative trait locus mapping Genetics 2009 181 3 1101 1113 10.1534/genetics.108.099556 19139143 PMC2651046

[B10] Rönnegård L Lee Y Hierarchical generalized linear models have a great potential in genetics and animal breeding Proc. WCGALP 2010 Leipzig, Germany

[B11] Lee Y Nelder JA Noh M H-likelihood: problems and solutions Statistical Computing 2007 17 49 55 10.1007/s11222-006-9006-7

[B12] Rönnegård L Shen X Alam M hglm: a package for fitting hierarchical generalized linear models The R Journal 2010 2 2 20 28

[B13] R Development Core Team R: A Language and Environment for Statistical Computing 2009 R Foundation for Statistical Computing, Vienna, Austria http://www.R-project.org

